# Assessment of Marital Satisfaction Among Spouses of Soldiers: The Example of Turkey

**DOI:** 10.1093/milmed/usad184

**Published:** 2023-05-19

**Authors:** Filiz Er, Elif Gokcearslan

**Affiliations:** Department of Socıal Work, Sınop Unıversıty, Faculty of Health Scıences, Sınop, Turkey 57100, Turkey; Department of Social Work, Ankara University Institute of Health Sciences, Ankara 06100, Turkey

## Abstract

**Introduction:**

Military personnel and their families face biopsychosocial risk factors due to frequent deployments, long and dangerous assignments, being away from home, not being able to spend time with their family, and adaptation to family life after returning from duty. These risks are among the factors affecting the marital satisfaction of military families.

**Materials and Methods:**

Our study population consists of 6 military spouses selected by the maximum sampling method, which the researchers reached using their resources. The research was conducted in Van Province between January and February 2021. The semi-structured interview form prepared by the researchers was used in the research designed with the qualitative research method. During the interviews, audio was recorded and transcribed.

**Results:**

With the findings obtained from the interviews, subthemes were formed by considering similar expressions used by the participants regarding their opinions under the main themes. The main themes that emerged in the research were the experience of being married to a soldier, relational satisfaction, the effect of duties on the relationship, and perception of social context. Considering all the results, it has been revealed that alongside long-term and far-from-home assignments due to the nature of military service, the military lifestyle determines the marital satisfaction of military spouses. Accordingly, it was observed that military spouses and families must be supported during the soldiers’ duties and complicated professional processes.

**Conclusions:**

This study reveals that long-term and far-from-home assignments due to military service impact the marital satisfaction. Accordingly, it was observed that military spouses and families must be supported during the soldiers’ duties and complicated professional processes.

## INTRODUCTION

Military personnel face the risk of many mental and social health problems due to their duties. These risks also apply to military families.^1–3^ Changes in society and family structure have brought various problems for military families. The increased involvement of women in business life and the number of married soldiers made it necessary to research the issues and needs of military families.^3,4^

Today, the role of social work has expanded in helping military personnel, veterans, and their families. Active-duty soldiers, veterans, and military families have recently dealt with various biopsychosocial problems. One of those problems is marital satisfaction in military families.^5,6^

Marital satisfaction is individuals’ subjective happiness in all aspects of their relationships.^7^ It has behavioral interconnectedness, fulfillment of needs, and emotional fondness.^7–9^ Many studies examined the processes related to marital satisfaction.^10–12^ Considering all these studies, it is seen that marital satisfaction, happiness, and harmony between spouses are affected by various factors in military families. The primary purpose here is to examine the marital satisfaction of Turkey’s military spouses. This will contribute to developing intervention plans, and social workers and other practitioners will gain new insights into understanding the subject.

## METHODS

### Research Method

The phenomenological method was used in this study. Phenomenological research is a qualitative research approach. This approach ignores biased assumptions about the phenomenon. Researcher’s experiences aim to gain in-depth information on how they understand these experiences. For this, it analyzes lived experiences.^13^

This study used a semi-structured interview form prepared by the researchers. All interviews were audio recorded and transcribed for analysis. The interview form consisted of 2 parts. The first part included questions directed toward the participant’s introduction and the second included open-ended questions about what affects the participant’s marital satisfaction. The second part consists of questions about the experience of being married to a soldier, relationships between spouses, and social networks.

### Participants

This Institutional Review Board–approved, voluntary, and anonymous qualitative research study was conducted in Turkey’s Van Province between January and February 2021. Our study population consists of 6 military spouses selected by the maximum sampling method. The participants with at least 2 years of marriage experience were considered. The participants were the spouses of the Gendarmerie General Command personnel. All of the participants were women.

The reason for focusing this study on the Gendarmerie General Command personnel is that the unit has more deployment and military assignment cycles. Wives of personnel from different statuses and ranks were selected. This selection was made to provide a more representative sample. Two participants are commissioned officers, 2 are non-commissioned officers, and 2 are expert gendarmerie spouses (expert gendarmerie is active-duty gendarmerie personnel serving in non-commissioned officer positions). The study started with 9 participants, but some could not participate because they were in different places of duty due to illness, assignment, or deployment. Voluntary consent forms were signed by the participants who volunteered to participate in the interviews.

The sample saturation point was taken to reference the study’s sample size.^14^ The saturation point means theoretical and thematic saturation in which no new information or theme is observed in the data obtained from case.^15^ Guest, Bunce, and Johnson, in their study with 60 interviewees, found that the essential elements of the themes were present in the first 6 interviews. In this small sample, predominant themes reached saturation in 4, 5, and 6 interviews.^16^

While in-depth information is provided by studies conducted with a small group like ours, some studies refer to the saturation point.^17–19^

### Data Collection

The research data were collected using face-to-face interview techniques in the participants’ homes and in interviews that lasted approximately 60 minutes. Researchers are social workers and women. The face-to-face interview ensures the data quality collected under the researcher’s supervision. Thus, corrections can be made during the data collection obtained from the participants. Additional questions can be asked immediately using this method. These advantages are not possible with other data collection methods, such as surveys or other methods.

### Data Analysis

A qualitative content analysis technique was used in our study.^20^ Transcribed interviews were analyzed with the MAXQDA 2020 software program. Repeated words and word sequences were searched by examining the data set, and codes were created. The common themes of the generated codes were focalized. The data were analyzed within the frame of the central theme, and subthemes were reached. With the creation of central themes and subthemes, a deeper understanding of the subject of the study was achieved.

## RESULTS

### The Sociodemographic Structure of Military Spouses and Findings on the Marriages

The introductory information form obtained the sociodemographic structure of military spouses and marriage information (Table I). The participants’ ages ranged from 18 to 38 years old. While 3 participants are high-school graduates, the education levels of the remaining participants are relatively lower. Four participants have reported that their monthly family income exceeds 10,000 Turkish liras. One of the participants married the person recommended by his family (arranged). None of the participants are working. None of the participants have helper/support at home.

**TABLE I.** Introductory Information about the Military Spouses

P. no	Duration of marriage	Number of kids	Monthly income (lira)	Way of marriage	Dating phase	Employment
1	≥9 years	1	<10,000	Flirt	4 years	No
2	<9 years	0	>10,000	Flirt	7 years	No
3	≥9 years	3	<10,000	Flirt	2 years	No
4	≥9 years	2	<10,000	Flirt	1 year	No
5	≥9 years	1	>10,000	Arranged	20 days	No
6	<9 years	2	<10,000	Flirt	8 months	No

### Findings Regarding the Evaluation of Marital Satisfaction of Military Spouses

Marital satisfaction evaluation of military spouses was obtained by using a semi-structured questionnaire. Participant interview analysis revealed main themes and a group of nested subthemes formed by considering related responses under these main themes. The main themes that emerged were the experience of being married to a soldier, relational satisfaction, the effect of duties on the relationship, and the perception of status relations. Accordingly, the main themes and subthemes that emerged are shown in Table II.

**TABLE II.** Code Diagram on the Evaluation of Military Spouses’ Marital Satisfaction

Main theme	Subtheme
Experience of being married to a soldier	Military marriage story Roles and division of labor Strategies for coping with marriage problems Women’s self-image
Relational satisfaction	Relationship happiness Sexual intimacy
Effect of duties on the relationship	Mutual attitude Adaptation
Perception of status relations	High expectations of core families Children’s experience Social support Lack of institutional help Suggestions for support

### First Main Theme: Experience of Being Married to a Soldier

#### Military marriage story (being both married and single)

It is emphasized that participants needed to learn the conditions of being married to a soldier at the beginning of their marriage process. The women’s families were hesitant about their marriage because of their concerns that the soldiers would work in distant cities.

G.5: I became aware that he was a soldier when I went to Sarıkamış (Turkey) at 18.

All of our study participants described the experience of being married to a soldier as complex, lonely, unplanned, uncertain, and unsupported and emphasized the military lifestyle. They said they tried to be empathetic toward their spouses and support them. Despite the difficulties, women described marriages with soldiers as instructive and transformative. They emphasized that they had evolved into strong women because they had to live in difficult conditions during this transformation process.

G.2: It was very informative; my perception changed; I learned that being a family means fighting together.

They emphasized that the issues they attach importance to regarding marital satisfaction are different from their civil counterparts and that it is essential that their spouses return from duty in good health or spend time at home for marital satisfaction.

G.5: It is great to be close to my husband.

All participants stated that being married to a soldier has an unplanned life. However, one participant emphasized that her satisfaction with her marriage was affected due to the conditions brought by marriage with a soldier and explained her situation as follows.

G.5: I never felt like I belonged to the marriage or the city we were in; we were always on edge.

One participant particularly emphasized the impact of missions on the marital experience.

G.3: He has been going on missions for 16 years; there is nothing that can ruin a marriage more.

#### Roles and division of labor (being both the man and the woman)

In the question about the distribution of duties and division of labor, all the participants emphasized that they took on the roles of both men and women in terms of roles and division of labor.

G.3: I am both the mother and the father; there is no division of labor.

G.6: I did everything by myself; I even went into labor because he was on a mission.

#### Strategies for coping with marriage problems (always staying on their right sides)

It was emphasized in the study that women produce various coping strategies for a solid and healthy continuation of marriage. All the participants stated that because of the empathy they established with their military spouses due to the difficulty of their duties, they tried not to impose too much responsibility on them when they returned from duty. Among those were trying to keep the relationship alive and warm by not leaving the city where the spouse works, even if the spouse does not come home, the effort to reduce the situations of moving and assignment, mutual suggestions, the constant communication while on duty, and the sharing of daily life events. Considering conditions such as the spouse’s stress, anger, and tiredness in the arising problems, the women stated that they are the party that compromises more by making sacrifices and trying to the last point of their strength.

G.6: It is a very stressful career, so naturally, this is reflected in domestic life; he does not reflect it on the family or does not scream at our child. You understand from his looks that something is wrong, and then you are forced to compromise in every way.

#### Women’s self-image (militarification of women at home)

When the study questioned how military wives should be, women’s perceptions of themselves were as strong, capable, and resourceful women due to their military lifestyle. Women used common expressions, especially when their spouses were on duty, were firm, upright, having healthy boundaries, loyal, protector of children, patient, instructive, and happy with little things.

### Second Main Theme: Relational Satisfaction

#### Relationship happiness (glad he married me)

In the study, most participants stated that they got more attached by facing challenging tasks, completing each other with their spouses, and progressing without breaking each other’s hearts. Despite all the difficulties, the spouses emphasized that they were satisfied with their marriage, and their satisfaction increased over time.

G.3: It is a great happiness to be in my spouse’s life.

G.5: I am glad he married me; I understand him.

One participant (G.1) stated that legal and health problems that occur in work life affect their marriage routines, creating problems and conflicts and affecting the marriage process and satisfaction. All the other participants mentioned that the core families of the soldiers had severe financial expectations. Women sought the support of their husbands’ peers and commanders to overcome these problems. Thus, practical ways were found to resolve conflicts in interpersonal relationships. Moreover, most women stated that this situation was valid for other military families. One of the participants summarized their experiences with the following expression.

G.5: Families should leave their soldier sons alone.

### Sexual Intimacy (Missing and Alienating)

All but one of the study participants emphasized that their sexual life was negatively affected. The negative impact is related to the adaptation process in returning from duty. It was noted that long-term and frequent separations and stress caused by duty affect the routine in sexual life and have more regular sexual activation after the adaptation process.

G.3: You suppress your feelings. Sometimes he would not come home for 7–8 months. Some distance was inevitable. We both learned to live without each other; we had not seen each other in nearly a year.

### Third Main Theme: Effect of Duties on the Relationship

#### Mutual attitude (appreciative men)

When the participants were questioned about how the duties were reflected in the spouses’ behavior, they mentioned that soldiers who went to duty very often and for a long time had positive and negative attitudes. Calmness, appreciation, and loyalty were positive; angry and aggressive attitudes were negative.

G.3: He reflects his stress onto us and orders us into the house.

On the other hand, women emphasized that they exhibited non-provocative, understanding, and accepting attitudes toward their spouses.

#### Adaptation (starting over and over)

All participants stated that their spouses, who returned from long-term duties, made plans for themselves, had difficulties keeping up with the order of the house, and acted inexperienced while participating in activities related to the family’s needs. They also emphasized repetitive routines such as leaving and returning home.

G.2: It is like starting over and over.

### Fourth Main Theme: Perception of Status Relations

#### High expectations of core families (seeing as a money source)

One of the original subthemes that emerged in the study, different from the international literature, is the high financial expectations of the core families of the soldiers. Except for one, the participants emphasized that families do not support their children in case of need and do not value their children enough.

G.5: I am the one that deals with all of the hardships when he is not here. I look after the kids, and I cook, etc. I am entitled to that money as much as he is, but families do not consider that.

#### Children’s experience (the father is both there and not there)

Participants mentioned that children exhibit sad and anxious moods and frequently experience physical ailments while their father is on duty. In addition, personality development is also affected. They mentioned that children perceive their fathers who return from duty as strangers, emulate other families, and grow up early.

G.1: I think the children make enormous sacrifices.

G.6: It affects him both mentally and physically. He does not eat and gets affected when he is with friends.

#### Social support (I am always alone)

Except for one, the participants emphasized that they tried to get social support from other military families. However, they stated that this support did not coincide with the social support provided by family and relatives. They noted that the core families did not understand the difficulties. In addition, the families of soldiers living in different cities emphasized that they could not receive social support from the local people and that the local people were reluctant to communicate.

G.3: Nobody understands; she summarizes the situation.

#### Lack of institutional help (I never got any)

All of the participants in our study emphasized the need for institutional support regarding the difficulties experienced. However, they stated that they did not receive any.

G.6: When (my spouse) went on duty for 60 or 45 days, nobody knocked on my door and asked me if I needed anything. That hurt me. It was something I expected from the military.

One participant expressed this angrily who wanted the institution to be more sensitive about family issues.

G.1: There was no support; they sometimes prevented it. A family should be able to have breakfast together on the weekends.

#### Suggestions for support (education)

Military spouses made suggestions, especially institutional support. The first suggestion was to organize training and seminars or create booklets on military life, housing culture, and etiquette rules. It was among the suggestions that families should be taken into account more for assignments or appointments. They suggested that experts hold meetings occasionally to protect their spouses’ mental health. Participants emphasized that they would like advice from experts who understand them.

G.1: It would be nice to have a psychologist or psychiatrist to talk to. There should have been someone experienced in the military that could understand what we were going through.

Another issue that military spouses suffer from is missed career opportunities. Spouses also emphasized the issue of providing employment opportunities for them, especially in the appointed places.

## DISCUSSION

Our study focused on the military lifestyle, experiences, and assignments away from home and investigated the factors affecting spouses’ marital satisfaction. Extended periods of assignments, resulting in being away from home and family, can potentially pose challenges to marital life. The perception of these random and indefinite assignments as a transformative lifestyle for women that profoundly affect women’s marital satisfaction is significant in terms of the implications of this study. Our study aims to examine marital satisfaction in military families. The results obtained from the research will contribute to developing plans related to the subject. These are the main contributions of the study.

Families of soldiers working in operational units assigned to combat areas experience many difficulties in daily routines. Studies have shown that war stress affects marital satisfaction, adaptation, and physical health.^21,22^ In the study, when the subthemes of the experience of marrying a soldier were examined, marriage to a soldier was perceived as a transformative and empowering experience. In this regard, Sherwood states that being a military spouse provides tremendous opportunities to grow under challenging conditions.^23^ The study determined that the situations that military spouses refer to regarding marital satisfaction differ from those of civilians.

Women feel an intense longing for their military spouses, which prolongs the marriage’s honeymoon period. It was understood that soldiers also developed a heightened sense of value for their wives. For such reasons, the marriage rates of military personnel differ from those of civilians of similar age. Studies have found that the marriage rate is higher in soldiers than in civilian counterparts.^24,25^ In the study, it was understood that the women assumed a supportive role in the marriage process with the help of the empathy they developed for their husbands’ complex duties. In this context, there are studies consistent with our study.^26^

The reorganization of family and personal roles can be another source of stress. Boss names this situation “ambiguous loss.”^27^ Parallel to this, in our study, it appeared that women evolved into male roles like it was the ordinary course of life; their expectations from their husbands decreased, and women continued to take almost all responsibilities even after their spouses returned from duty. Roles and responsibilities continued as they did when men were on duty. Both men and women have embraced their roles in the appropriate distribution of roles and responsibilities.

Women contribute to the sacred patriotic duty by taking responsibility at home and enduring all difficulties. During assignments, women may experience severe concerns about their spouses. Therefore, they can idealize their relationships. In addition, it was found in a study that frequent communication during duty provides high marital satisfaction.^28^ In our study, women developed coping strategies such as frequently communicating at work and being proud of their spouses. This situation is consistent with previous studies.^29^

Notably, women do not display a critical and accusatory attitude toward their husbands regarding the problems arising from their duties. On the contrary, they acted with an accepting and resilient attitude. This attitude indicates that women are primarily responsible for establishing and maintaining marriage in traditional Turkish society. This situation is consistent with previous studies conducted.^8^

As it can be understood from the common emphases used by women in the study, they clearly described the image of women who become soldiers at home, who are physically and mentally strong, and support their husbands in return for their military spouses who are fighting at the front. These women should be discreet women who can organize many things simultaneously and are self-sufficient for their children. What draws attention here is that, in the context of gender roles, women who fight on the home front are more accepted than naïve and fragile women who do not interfere in men’s affairs. At this point, women, one of the 2 strong sides of the romantic relationship, seem more robust when balancing the relationship with their military spouses. Moreover, this situation becomes easier to cope with when a sacred duty such as homeland protection is considered.

In a study, interpersonal processes in solving marital conflicts and marital problems were emphasized to understand the variability in marital satisfaction comprehensively.^8^ While challenging assignments and the military lifestyle affect marital satisfaction, women tend to protect their military spouses and marriages in a way that even core families do not understand. The interview point is that the women are happy with their wedding even though the difficulties continue. In a study, US military personnel had similar relationship difficulties, but the relationship satisfaction rate was 86.9%.^12^

The results of the sexual intimacy between the spouses were evaluated. It was revealed that the sexual life of almost all participants was affected due to long separations. An adaptation period may be required to reestablish the emotional and physical bond. The study results are parallel to the results of our study.^30,31^

One of the main themes that emerged in the study is that frequent and prolonged assignments affect the behavior of women and soldiers positively and negatively. Soldiers perceive their wives as the protectors of the families they left behind, showing gratitude and affection to them. Women are aware of the source of their interest and are happy with it. However, difficulties such as commitment to duty and friends, inability to adapt to domestic functioning, and reflecting their stress at home can be perceived as negative behaviors. In this case, it was understood that women took steps not to escalate the conflict. In the study, Britt et al. reported that individuals primarily devoted to work have lower marital satisfaction levels.^32^

It was understood that there were difficulties in getting together and sharing the roles after the soldier returned from duty. Another study revealed that returning from duty is the most stressful part for many families.^2^ Our study determined that the soldiers experienced difficulties returning and adapting to the prior home life due to the highly intense military work while away. It may take time for the soldier to adapt to the role of husband and father. It takes time for women to get used to having a spouse. Previous studies on this subject are consistent with our research.^33–35^

One of the original subthemes that can be considered specific to Turkish culture is the high financial expectation of the soldiers’ core families. Women develop strategies against core families. This strategy is the most apparent factor affecting marital satisfaction. This situation also caused discredit toward core families. This situation has emerged as another area of loneliness for military families who feel lonely and unsupported in different cities. Parallel to our study, the importance of individual and organizational support was emphasized in previous studies.^36,37^ According to our study results, military spouses with similar concerns can assume the role of the core family by developing friendships in military housing. The result of the study on the subject is consistent with our study.^34,37^ Our study showed that prolonged and frequent military assignments, uncertainties, and unplanned arrivals and departures to children’s duties, assignments, and responsibilities create difficulties that affect marital satisfaction. Previous studies have revealed the importance of institutional support.^34,38^

In international literature, symptoms such as sadness, emotionality, insomnia, aggression, irritability, depression, and decreased achievement have been reported in military children and a tendency to physical diseases.^34,39,40^ In our study, although this situation affects marital satisfaction, children became sensitive to these reactions and were supportive.

There are some limitations of our study. The study results cannot be generalized to the entire military system. Similarly, military personnel of lower rank is deployed or assigned more frequently than those of higher rank.

## CONCLUSION

It was observed in participant responses that military spouses and families must be supported during the soldiers’ duties and complicated professional processes. This necessarily includes social support that focuses on the whole family to increase marital satisfaction and adapt family relations due to changing military service conditions.

Increasing the marital satisfaction of military spouses is a complex issue that requires a multidimensional approach. Our research has shown that military spouses face unique challenges, such as frequent relocation, prolonged separation from their spouses, and the stress associated with their spouse’s deployment.

Providing social support and resources to military spouses is critical. By providing resources to help alleviate stressors, military spouses can experience increased marital satisfaction. Effective conflict resolution strategies should be developed. Military couples should strengthen their communication skills. The unique contributions of military spouses to their families and communities should be recognized and supported.

The responses in this study may provide important data for future research and programming. Some research topics are as follows:

- Developing practices to support military spouses’ unique contributions to society through the armed forces;
- Encouraging the participation of military spouses in various decision-making processes;
- Providing opportunities for personal and professional development.

## Data Availability

The data that support the findings of this study are available on request from the corresponding author. All data is freely accessible.

## CLINICAL TRIAL REGISTRATION: IDENTIFIER

No Clinical Trial.

## INSTITUTIONAL ANIMAL CARE AND USE COMMITTEE (IACUC)

No animal.

## INSTITUTIONAL CLEARANCE

Institutional clearance approved.
